# Recommendations from the European Commission Initiative on Breast Cancer for multigene testing to guide the use of adjuvant chemotherapy in patients with early breast cancer, hormone receptor positive, HER-2 negative

**DOI:** 10.1038/s41416-020-01247-z

**Published:** 2021-02-18

**Authors:** Paolo Giorgi Rossi, Annette Lebeau, Carlos Canelo-Aybar, Zuleika Saz-Parkinson, Cecily Quinn, Miranda Langendam, Helen Mcgarrigle, Sue Warman, David Rigau, Pablo Alonso-Coello, Mireille Broeders, Axel Graewingholt, Margarita Posso, Stephen Duffy, Holger J. Schünemann, the ECIBC Contributor Group, Mariangela Autelitano, Bettina Borisch, Xavier Castells, Edoardo Colzani, Jan Daneš, Patricia Fitzpatrick, Livia Giordano, Solveig Hofvind, Lydia Ioannidou-Mouzaka, Susan Knox, Lennarth Nystrom, Elena Parmelli, Elsa Perez, Alberto Torresin, Ruben Van Engen, Cary Van Landsveld-Verhoeven, Ken Young

**Affiliations:** 1 Azienda Unità Sanitaria Locale—IRCCS di Reggio Emilia, Reggio Emilia, Italy; 2 Department of Pathology, University Medical Center Hamburg-Eppendorf, Hamburg, Germany; 3 Iberoamerican Cochrane Center, Biomedical Research Institute (IIB Sant Pau-CIBERESP), Barcelona, Spain; 4 Department of Paediatrics, Obstetrics and Gynaecology, Preventive Medicine, and Public Health, PhD Programme in Methodology of Biomedical Research and Public Health, Universitat Autònoma de Barcelona, Bellaterra, Spain; 5 European Commission, Joint Research Centre (JRC), Ispra, Italy; 6 Instituto de Salud Carlos III, Health Technology Assessment Agency, Avenida Monforte de Lemos 5, Madrid, Spain; 7 St. Vincent’s University Hospital, Dublin, Ireland; 8 Department of Clinical Epidemiology, Biostatistics and Bioinformatics, Amsterdam UMC, University of Amsterdam, Amsterdam Public Health Institute, Amsterdam, The Netherlands; 9 Cardiff and Vale UHB - General Surgery, Cardiff, UK; 10 Havyatt Lodge, Havyatt Road, Langford, North Somerset UK; 11 Department for Health Evidence, Radboud University Medical Center, Nijmegen, the Netherlands; 12 Dutch Expert Centre for Screening, Nijmegen, the Netherlands; 13 Radiologie am Theater, Paderborn, NRW Germany; 14 Department of Epidemiology and Evaluation, IMIM (Hospital del Mar Medical Research Institute), Barcelona, Spain; 15 Research Network on Health Services in Chronic Diseases (REDISSEC), Barcelona, Spain; 16 Centre for Cancer Prevention, Queen Mary University of London, Charterhouse Square, London, UK; 17 Michael G. DeGroote Cochrane Canada and McGRADE Centres; Department of Health Research Methods, Evidence and Impact, McMaster University Health Sciences Centre, Hamilton, Ontario, Canada; 18 Cancer Registry of Milan, Milan, Italy; 19 Institute of Global Health. University of Geneva, Geneva, Switzerland; 20 IMIM, Barcelona, Spain; 21 European Centre for Disease Control and prevention (ECDC), Solna, Sweden; 22 Charles University in Prague, Prague, Czech Republic; 23 National Screening Service, Dublin, Ireland; 24 CPO-Piedmont - AOU Citta` della Salute e della Scienza, Torino, Italy; 25 Cancer Registry of Norway, Oslo, Norway; 26 University of Athens Medical School, Athens, Greece; 27 Europa Donna, Milan, Italy; 28 Umea University, Umea, Sweden; 29 European Commission, Joint Research Centre, Ispra, Italy; 30 University Hospital Dr. Josep Trueta, Girona, Spain; 31 Ospedale Niguarda Ca’ Granda, Milan, Italy; 32 Dutch Reference Centre for Screening, Nijmegen, The Netherlands; 33 National Coordinating Centre for the Physics of Mammography, Guildford, UK

**Keywords:** Prognostic markers, Breast cancer

## Abstract

**Background:**

Predicting the risk of recurrence and response to chemotherapy in women with early breast cancer is crucial to optimise adjuvant treatment. Despite the common practice of using multigene tests to predict recurrence, existing recommendations are inconsistent. Our aim was to formulate healthcare recommendations for the question *“Should multigene tests be used in women who have early invasive breast cancer, hormone receptor-positive, HER2-negative, to guide the use of adjuvant chemotherapy?”*

**Methods:**

The European Commission Initiative on Breast Cancer (ECIBC) Guidelines Development Group (GDG), a multidisciplinary guideline panel including experts and three patients, developed recommendations informed by systematic reviews of the evidence. Grading of Recommendations Assessment, Development and Evaluation (GRADE) Evidence to Decision frameworks were used. Four multigene tests were evaluated: the 21-gene recurrence score (21-RS), the 70-gene signature (70-GS), the PAM50 risk of recurrence score (PAM50-RORS), and the 12-gene molecular score (12-MS).

**Results:**

Five studies (2 marker-based design RCTs, two treatment interaction design RCTs and 1 pooled individual data analysis from observational studies) were included; no eligible studies on PAM50-RORS or 12-MS were identified and the GDG did not formulate recommendations for these tests.

**Conclusions:**

The ECIBC GDG suggests the use of the 21-RS for lymph node-negative women (conditional recommendation, very low certainty of evidence), recognising that benefits are probably larger in women at high risk of recurrence based on clinical characteristics. The ECIBC GDG suggests the use of the 70-GS for women at high clinical risk (conditional recommendation, low certainty of evidence), and recommends not using 70-GS in women at low clinical risk (strong recommendation, low certainty of evidence).

## Background

Breast cancer is the most frequently diagnosed cancer among women.^1^ In the European Union, including UK, 404,920 women were diagnosed with breast cancer and 98,755 died because of this disease in 2018.^2^ Hormone receptor (HoR)-positive (i.e. oestrogen receptor (ER)- and/or progesterone receptor (PR)-positive), human epidermal growth factor receptor 2 (HER2)-negative breast cancer represents about 70% of breast cancer diagnosed in western countries.^3^ At the time of diagnosis, around 60% of this type of cancer has not spread to lymph nodes,^3^ and approximately 15% of these women will develop a recurrence within 10 years if treated with adjuvant endocrine therapy alone.^4,5^ The risk of recurrence could be reduced by the addition of chemotherapy.^5^ However, given the relatively low risk of recurrence and the partial effectiveness of chemotherapy in these women, most would be over-treated if all received chemotherapy. The same rationale would apply to women with HoR-positive, HER2-negative invasive breast cancer with 1–3 positive lymph nodes.^6^ Several prognostic factors, including clinical-pathological features such as age, tumour size, percentage of ER- and PR-positive cells as well as Ki67-index,^7–9^ predict the risk of recurrence and can help identify women who would benefit the most from chemotherapy. Although these factors have been shown to discriminate different prognostic groups, they showed no or minimal predictive value on the response to chemotherapy.^10^

In the last 15 years, different tests have been developed to stratify patients with early breast cancer into different risk of recurrence groups by analysing the activity of various genes. Although these multigene tests use diverse techniques (RT-PCR, microarray, and others) and diverse target gene combinations, they all focus on genes involved in cell proliferation. The tests provide recurrence risk profiles categorised in different ways. Some tests have been explicitly proposed to provide additional information to clinical-pathological features, as the 12-MS and the PAM50-RORS. Based on the results of the MINDACT trial,^6^ the application of the 70-GS also takes into account clinical prognostic characteristics, while the 21-RS has been proposed to substitute clinical risk-based treatment decisions.

The European Commission Initiative on Breast Cancer (ECIBC) aims to provide evidence-based recommendations for screening and diagnosis of breast cancer.^11^ The Guidelines Development Group (GDG) of the ECIBC prioritised a clinical question on the use of multigene tests to guide the use of adjuvant chemotherapy in HoR-positive, HER2-negative and lymph node-negative or up to 3 lymph nodes-positive invasive breast cancer. Four multigene tests, used to stratify women with breast cancer into different groups according to recurrence risk,^12–17^ are included in the clinical question (Supplementary Table 1): 21-RS (Oncotype DX, Genomic Health Inc), 12-MS (EndoPredict, Myriad Genetics Inc), PAM50-RORS (Prosigna test, NanoString Technologies Inc.), and 70-GS (MammaPrint; Agendia Inc). Direct comparison between different tests is beyond the scope of these recommendations.

## Methods

### Structured question and outcome prioritisation

The clinical question “*Should multigene tests be used in patients who have HoR-positive, HER-2 negative, lymph node-negative or up to 3 lymph nodes-positive invasive breast cancer to guide the use of adjuvant chemotherapy*” was structured following the Population, Intervention, Comparison and Outcomes (PICO) format (Table 1). The outcomes were also prioritised by the GDG using a nine-point scale (7 to 9 critical; 4 to 6 important; 1 to 3 of limited importance), as suggested by the Grading of Recommendations Assessment, Development and Evaluation (GRADE) approach.^11,18^ The GDG decided not to attempt any head-to-head comparisons between the different tests.

**Table 1:** Structured clinical questions.

Population	Intervention	Comparison	Outcomes
Patients with hormone receptor-positive, HER2- negative, lymph node-negative or up to 3 lymph nodes-positive invasive breast cancer.	70-gene signature to decide chemotherapy: women with low clinical risk do not receive chemotherapy independently from 70-gene signature; women with high clinical risk will receive chemotherapy only if at high genomic risk	• According to the considered clinical trial designs the comparison would be either: • All patients receive chemotherapy • All patients with low clinical risk do not receive chemotherapy and those with high clinical risk subgroup receive chemotherapy. Direct comparisons between the different multigene tests were not performed.	• Overall survival • Disease-free survival • Adverse effects of drugs • Quality of life.
	21-gene recurrence score to decide chemotherapy, in two alternative scenarios: • Women with low or intermediate genomic risk will not receive chemotherapy, women with high genomic risk will; • Only women with high clinical risk will be tested for genomic risk and receive chemotherapy if genomic risk is high; women with low clinical risk will not receive chemotherapy.		

Initially PICOs covered four multigene tests, but two of them, the PAM50 risk of recurrence score (RORS) and 12-gene molecular score (12-MS), were excluded because of study selection criteria. These selection criteria allowed no direct comparison between the different multigene tests.

### Systematic review

#### Data sources and searches

MEDLINE (May 2018), EMBASE (May 2018) and CENTRAL (May 2018) databases were searched, using pre-defined algorithms, for both systematic reviews and individual studies (Supplementary Table 2); this original search was continuously run up to October 2018. Lists of references of the included studies were reviewed and members of the GDG were requested to provide additional studies.

#### Study selection

Randomised controlled trials (RCT) and cohort studies (including pooled analyses of studies), either from prospective or retrospective analysis, of stored specimen samples were included as long as they applied any of the four tests as predictive markers for guiding the use of adjuvant chemotherapy (Supplementary Fig. 1)

A predictive marker identifies the differential benefit of a treatment based on the marker status. Thus, we included the following assessment approaches: (a) *Marker-based strategy design*: patients are assigned to a treatment arm depending on whether they received treatment (i.e. endocrine therapy or endocrine plus chemotherapy) according to the test results or according to usual clinical practice. The predictive value is assessed by comparing the outcomes from the testing-based arm versus the non-testing arm; (b) *Treatment interaction design*: patients are divided into groups based on the marker status (i.e. high and low marker status). Then they are allocated to receive endocrine therapy or endocrine treatment plus chemotherapy. The predictive value is assessed by observing the relative efficacy of treatment differences between marker status and treatment assignments.

Studies that only reported prognosis data based on marker status (without considering differential treatment effect), individual observational studies, abstracts or conference communications not published as full text articles, and articles published in a language other than English were excluded.

With respect to economic evidence, cost-utility, cost-benefit, and cost-consequences, analyses were included if conducted within clinical trials, as well as observational and modelling studies, published in English during the last decade (Supplementary Table 3). After a calibration process, each reviewer (CCA and KP) assessed titles and abstracts for eligibility. Subsequently, two reviewers (CCA and KP), independently, reviewed the full text of all the pre-selected references. Discrepancies were solved either by consensus or with the help of a third reviewer (DR) (Supplementary Fig. 2 and Supplementary Table 4).

#### Data extraction and risk of bias assessment

Two reviewers (CCA and KP) independently assessed risk of bias and extracted the following information from each study: first author, year of publication, country, study design, inclusion and exclusion criteria, number of patients, age, participants’ characteristics and prioritised outcomes.

The risk of bias of the included RCTs was assessed using the Cochrane Risk of Bias tool for randomised trials.^19^ Cohort studies were assessed with the “Risk Of Bias In Non-randomised Studies - of Interventions-I” (ROBINS-I) tool.^20^ For economic evaluations, one reviewer (MP) screened the search results and used the NICE methodology checklist to assess applicability and methodological limitations.^21^ Studies with poor applicability and/or high risk of bias were excluded (Supplementary Fig. 2).

#### Data analysis

Descriptive statistics were used to summarise the characteristics of the included patients across studies. The effect measures for prioritised outcomes and their corresponding 95% confidence intervals (CIs) were reported as presented in individual studies.

#### Certainty of the evidence

The certainty of evidence per outcome and overall certainty was rated using the GRADE approach. For each recommendation, the GDG received a Summary of Findings (SoF) table and a first draft of an evidence to decision framework (EtD).^22^

### Comparison scenarios and modelling

A simple *deterministic decision tree model* without discounting was built by PGR with input from the rest of the GDG to estimate the downstream consequences of testing patients with the multigene tests versus different scenarios of usual care (Supplementary Fig. 3). For the 21-RS, the general model assumptions were: the population of eligible women was divided into the three risk groups, as reported in the TAILORx trial at recruitment until 2008 (14% low risk of recurrence, 68% intermediate, 18% high).^23^ Rate of events, observed in the RCTs, were applied to the simulated usual care arms. Clinical risk of recurrence was classified as low and high according to the modified AdjuvantOnline!Score.^24,25^ Results are based on a fixed observation time of 10 years.

Two strategies for implementing the multigene test to guide the use of adjuvant chemotherapy were considered as interventions (Supplementary Fig. 3a):

1. All women would undergo multigene testing and adjuvant chemotherapy would be given accordingly (only to those classified in the high genomic risk group, i.e. with a score ≥26).
2. Only women with high clinical risk would undergo multigene testing, and only those with high genomic risk would receive chemotherapy. Women with low clinical risk would not receive it.

Two scenarios were considered as usual care comparators (“C” of the PICO framework) (Supplementary Fig. 3b):

1. All women would be referred to adjuvant chemotherapy (assuming 18.4% would not comply, i.e. the proportion of women not receiving chemotherapy among those assigned to the treatment arm in TAILORx).^23^
2. Women would receive adjuvant chemotherapy only if the clinical risk is high. The model assumes that women with low clinical and high genomic risk, as well as those with low clinical and low genomic risk, do not benefit from adjuvant chemotherapy. For sensitivity analyses, in women of low clinical and high genomic risk, two different assumptions were used to estimate the benefits: (a) The advantage of receiving adjuvant chemotherapy is equivalent to that observed in the MINDACT trial^6^ at five years, and the effect is maintained at 10 years; (b) the advantage from adjuvant chemotherapy is equivalent to that observed by Paik and colleagues^26^ for all women at high genomic risk, independent of their clinical risk. Distributions of the clinical risk within the multigene risk strata are those reported in the TAILORx trial.^23^

For the 70-GS, we focused only on comparing strategy 2 with scenario 2 in which women at high clinical risk would be tested and/or treated, because the evidence from the MINDACT trial indicates a very small benefit, if any, from adjuvant chemotherapy in women with low clinical risk, independent of their genomic risk.^6^

### Evidence to decision and recommendation formulation

The process the ECIBC GDG used to formulate recommendations has been described in a dedicated article published elsewhere.^11^ In brief, a subgroup of GDG members including experts on the topic and an informed patient (the so-called PICO responsible unit), took primary responsibility for the review and completion of the first draft of the SoF tables and the EtD frameworks, conducted initially by the systematic review team. The frameworks were used in the meetings to help the complete GDG formulate the recommendations. Subsequently they were reviewed by a technical team from the Joint Research Centre, the PICO responsible unit and the systematic review team. Finally, the recommendations and frameworks were approved by the GDG.

## Results

### Included studies

We included five studies (Supplementary Fig. 1): two RCTs,^6,23^ two secondary analyses of stored tissue blocks collected from former parent clinical trials^26,27^ and one pooled analysis of observational studies^12^ from four previously reported validation studies, including unpublished data (Supplementary Table 5).^28–30^

### 21 gene recurrence score

#### Treatment interaction design studies

Paik and colleagues^26^ provided estimates for distant recurrence free survival in patients with lymph node-negative breast cancer stratified into three levels of the 21-RS risk groups. Adding chemotherapy to endocrine therapy, compared to endocrine therapy alone, may have a different effect on recurrence across groups, i.e. a larger effect in women with higher 21-RS, but the evidence is very uncertain: hazard ratio (HR) of 1.31 (95% CI 0.46–3.78), 0.61 (95% CI 0.24–1.59) and 0.26 (95% CI 0.13–0.53) in low, intermediate and high risk groups, respectively (Supplementary Table 6).

Albain and colleagues^27^ included stored tumour specimens for genomic testing of postmenopausal women with HoR-positive, node-positive breast cancer. They performed an analysis adjusted by the number of positive nodes that suggests no benefit for chemotherapy on disease free survival (DFS) in the low genomic risk group (HR = 1.02; 95% CI 0.54–1.93) and a potential advantage in the high genomic risk (HR = 0.59, 95% CI 0.35–1.01) (Supplementary Table 6). The authors refer similar results for overall survival (OS).

#### Marker-based strategy

Sparano and colleagues^23^ provided results for several disease-free survival (DFS)-related outcomes among women with an intermediate genomic risk group (11 to 26 risk score) allocated to either endocrine therapy alone or chemotherapy plus endocrine therapy. The as-treated results suggest little to no difference in the risk of recurrence with chemotherapy plus endocrine therapy for invasive DFS (HR 1.14; 95% CI 0.99–1.31). For distant metastases, local recurrence and OS, similar results were observed (Supplementary Table 6).

### 70-GS

#### Treatment interaction design studies

Knauer and colleagues^12^ described results from a pooled database analysis with a median follow-up time of 7.1 years. Patients with a low and high genomic risk who received chemotherapy may have a lower risk of recurrence than those with endocrine therapy alone, but the evidence is very uncertain (HR 0.26; 95% CI 0.03–2.02 and HR 0.35; 95% CI 0.17–0.71, respectively). The results for mortality were consistent with the observed pattern of the risk of recurrence but the evidence was also very uncertain (HR 0.58; 95% CI 0.07–4.98; HR 0.21; 95%CI 0.07–0.59, respectively) (Supplementary Table 7).

#### Marker-based strategy

Cardoso and colleagues^6^ reported DFS and OS among patients in the clinical/genomic discordant-risk groups which were allocated to receive either chemotherapy in addition to endocrine therapy or endocrine therapy alone.

Women with high clinical risk and low genomic risk may have an increase of DFS (HR 0.64; 95% CI 0.43–0.95), of distant metastases free survival (HR 0.65; 95% CI 0.38–1.10) and of OS (HR 0.63; 95% CI 0.29–1.37) (Supplementary Table 7). The group of low clinical risk and high genomic risk showed imprecise effects and uncertain evidence for DFS (HR 0.74; 95% CI 0.40–1.39), distant metastases free survival (HR 0.90; 95% CI 0.40–2.01) and OS (HR 0.72; 95% CI 0.23–2.24).^6^

### Modelling for predicting impact of testing on patient’s outcomes

Depending on the different treatment scenarios (all women are referred to chemotherapy or only women with high clinical risk are treated with chemotherapy) and genetic testing strategies (genetic testing carried out in all women or testing only those with high clinical risk), the number of women who avoid chemotherapy by using the 21-RS would change from more than 600 to about 200. Survival outcomes did not change substantially (Table 2) for the 21-RS based on the benefits from adding chemotherapy in the MINDACT trial.^6^ However, on the assumption that all women with high genomic score would obtain the same benefits from adding chemotherapy as observed by Paik and colleagues,^26^ independently from their clinical risk, the intervention could potentially prevent 37 distant metastases compared to a scenario in which only women with high clinical risk would be treated with chemotherapy.

**Table 2:** Anticipated outcomes for different comparisons between the 21-gene recurrence score testing strategies (interventions) and comparator scenarios (no testing) per 1000 women with hormone receptor-positive, HER2-negative, lymph node-negative invasive breast cancer.

Outcome	Intervention strategy 1^a^	Comparator scenario 1^b^	Absolute difference	Intervention strategy 1^a^	Comparator scenario 2^b^	Absolute difference	Intervention strategy 2^a^	Comparator scenario 2^b^	Absolute difference
Number of Chemotherapy	180	820	−636	180	314	−134	103	314	−211
Invasive disease recurrence	80	78	2	80	79	−1	80	79	−1
Distant recurrence	27	26	1	28	27	0	27	27	0
Local and distant recurrence	39	38	1	39	39	0	39	39	0
Deaths	24	23	1	24	24	0	24	24	0

^a^According to Supplementary Fig. 3a.

^b^According to Supplementary Fig. 3b.

On the other hand, we considered for the 70-GS a two-step strategy according to the results of the MINDACT trial, testing only women with high clinical risk.^6^ Consequently, the only scenario considered was one in which only high-risk women would receive chemotherapy (Table 3). The use of the 70-GS would result in an avoidance of chemotherapy in about 230 women out of 1000 associated with small increase of recurrences.

**Table 3:** Anticipated outcomes for the comparison between the 70-gene signature assay testing strategy (intervention) and comparator scenario (no testing) per 1000 women with hormone receptor-positive, HER2-negative, lymph node-negative or up to 3 lymph nodes-positive invasive breast cancer.

Outcome	Intervention strategy 2^a^	Comparator scenario 2^b^	Absolute difference
Treated women	270	501.4	−232
Distant disease	53	48	4.5
Disease free	100	93	7
Deaths	30	26	3.5

^a^According to supplementary Fig. 3a.

^b^According to supplementary Fig. 3b.

### Results from the systematic review of economic evidence

From the primary literature search and from the two identified systematic reviews,^31,32^ 12 cost-effectiveness evaluations were identified (Supplementary Fig. 2 and Supplementary Table 4).^33–44^ The GDG agreed that these economic evaluations used models that were not directly applicable to the clinical question of interest. Therefore, cost-effectiveness was evaluated considering the benefits and harms estimated using the GDG’s ad-hoc model described above (Supplementary Fig. 3) and the costs reported by the studies included in the literature review. Eight studies reported costs for the use of the 21-RS in women with negative lymph nodes,^33–41^ whereas three reported costs for women with up to 3 positive lymph nodes.^42–44^ The reported costs of the 21-RS were EUR 3180 per patient in five out of the 11 studies included. These costs did not show significant differences between countries or over time. For the 70-GS, two studies reported costs of EUR 3153 per patient for the use of this assay in women with negative lymph nodes.^40,41^

**Table 4:** Judgements by the Guideline Development Group (GDG) in Evidence to decision framework for the question: Should multigene tests be used in patients who have hormone receptor-positive, HER-2 negative, lymph node-negative or up to 3 lymph nodes-positive invasive breast cancer to guide the use of adjuvant chemotherapy?

	21-gene recurrence score (limited to women with negative lymph nodes)	70-gene signature assay	
		Low clinical risk	High clinical risk
Problem	Yes	Yes	Yes
Desirable effects	Large	Trivial	Large
Undesirable effects	Trivial	Trivial	Small
Certainty of evidence	Very low	Low	Low
Values	Probably no important uncertainty or variability	Probably no important uncertainty or variability	Probably no important uncertainty or variability
Balance of effects	Probably favours the intervention	Favours the comparison	Probably favours the intervention
Resources required	Large costs	Large costs	Large savings
Certainty of evidence of required resources	Very low	Very low	Very low
Cost effectiveness	No included studies	No included studies	No included studies
Equity	Probably reduced	Probably reduced	Probably reduced
Acceptability	Varies	Varies	Varies
Feasibility	Varies	Varies	Varies
Final recommendation	Conditional in favour of the intervention	Strong against intervention	Conditional in favour of the intervention

### Certainty of evidence

The overall certainty of the evidence was rated as low to very low. The main concerns across studies were risk of bias, indirectness of trial populations and imprecision (Supplementary Tables 6 and 7). The evidence was also downgraded for indirectness due to the assumptions used for the model that implied the use of evidence from one population in another population and from different duration of follow-up across studies.

### Evidence to decision frameworks

#### 21-RS

The GDG judged the anticipated desirable effects (i.e. the avoided chemotherapy treatments) of using the test to guide chemotherapy to be large and the undesirable effects (i.e. increase in recurrence) trivial, with very low certainty of the evidence. The costs were considered large, though no cost-effectiveness study was included. A negative impact on equity was considered a potential concern (Table 4).

For women with HoR-positive, HER2-negative, node-negative invasive breast cancer, the ECIBC GDG suggests the use of the 21-RS to guide the use of chemotherapy (conditional recommendation, very low certainty of the evidence, Table 5). The recommendation is conditional because the certainty of evidence was very low and the downstream consequences of avoiding chemotherapy were not quantified, thus making the balance of benefits and harms difficult to determine, together with the large resource (costs) requirements (Table 4, see also https://healthcare-quality.jrc.ec.europa.eu/sites/default/files/Guidelines/EtDs/Updated/ECIBC_GLs_EtD_21_gene_recurrence_score.pdf).

**Table 5:** European Commission Initiative on Breast Cancer guidelines development group recommendations on the use of multigene tests to guide the use of adjuvant chemotherapy in patients with early breast cancer, hormone receptor-positive, HER-2 negative.

	21-gene recurrence score	70-gene signature	
		Women with low clinical risk^a^	Women with high clinical risk^a^
Recommendation	For women with hormone receptor-positive, HER2-negative, lymph node-negative invasive breast cancer, the ECIBC’s Guidelines Development Group (GDG) suggests using the 21-gene recurrence score to guide the use of chemotherapy.	For women with hormone receptor-positive, HER2-negative, lymph node-negative or up to 3 lymph nodes-positive invasive breast cancer at low clinical risk, the ECIBC’s Guidelines Development Group (GDG) recommends not using the 70-gene signature test to guide the use of chemotherapy.	For women with hormone receptor-positive, HER2-negative, lymph node-negative or up to 3 lymph nodes-positive invasive breast cancer at high clinical risk, the ECIBC’s Guidelines Development Group (GDG) suggests using the 70-gene signature test to guide the use of chemotherapy.
Strength	Conditional recommendation for the intervention Very low certainty of the evidence	Strong recommendation against the intervention Low certainty of the evidence	Conditional recommendation for the intervention Low certainty of the evidence
Sub-group considerations	The GDG did not consider women with node-positive invasive breast cancer to be included in this recommendation. Women with high clinical risk^a^ and low genomic risk (larger tumour diameter and higher grade) may experience larger net desirable consequences and provide a better cost-benefit profile. Women with low clinical risk^a^ and high genomic risk may experience smaller or no net desirable consequences. Indirect evidence from other gene based testing (e.g. 70-gene signature) supports that conclusion.	The proportion of women with 2 or 3 node-positive breast cancer was small, so the results may be less clear in this subgroup.	The proportion of women with 2 or 3 node-positive breast cancer was small, so the results may be less clear in this subgroup.

^a^For definitions of low and high clinical risk, see Supplementary Table 8.

**Table 6:** Synopsis of American Society of Clinical Oncology (ASCO)^59–61^ and the UK National Institute for Health and Care Excellence (NICE)^55^ recommendations on 21-gene recurrence score and 70-gene signature assay in hormone receptor-positive, HER2-negative, lymph node-negative or up to 3 nodes-positive invasive breast cancer.

	ASCO^58,60^	NICE^54^
*21-gene recurrence score*		
Low clinical risk	Strong recommendation	
High clinical risk	Strong recommendation	Recommended if: clinical risk is “intermediate” according to the PREDICT tool^53^ or the Nottingham Prognostic Index, i.e. the additional benefit of chemotherapy is between 3 and 5% increase in survival, the decision on the therapy will depend on the test result, the test is provided at reduced price.
Negative lymph nodes	Strong recommendation	Recommendation applies to both lymph node-negative patients and lymph node-positive patients, restricted to micro-metastases
1 to 3 positive lymph nodes	Not recommended	

	ASCO^60^	NICE^55^
*70-gene signature assay*		
Low clinical risk	Strong against	Recommendation against because not cost effective
High clinical risk	Strong in favour	
Negative lymph nodes	Strong in favour	
1 to 3 positive lymph nodes	Moderate in favour	

**Fig. 1:**
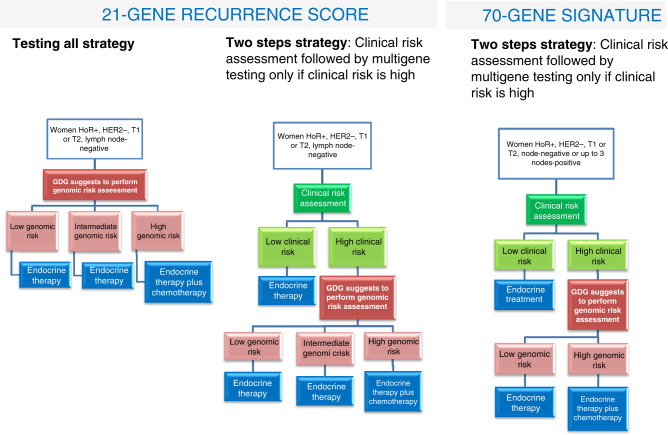
Flow charts reporting the possible uses of 21-gene recurrence score and for the 70-gene signature test to guide the use of adjuvant chemotherapy in patients with early invasive breast cancer, hormone receptor-positive, HER-2 negative. Two strategies are proposed for 21-gene recurrence score, the first in which all women are tested for genomic risk assessment and treated accordingly, the second in which only women with high clinical risk are tested for genomic assessment, while those at low clinical risk are referred to endocrine therapy alone without genomic risk assessment. According to sub-group considerations reported by the GDG, the latter strategy is probably more cost effective and women might experience larger net desirable consequences. For the 70-gene signature only a two-step strategy is proposed where only women at high clinical risk are tested for genomic risk; testing women at low clinical risk is not recommended.

The GDG did not consider women with node-positive invasive breast cancer in this recommendation, because they were not included in the TAILORx trial,^23^ the main source for model parameters. The GDG also stated that sub-populations with high clinical risk (defined according to AdjuvantOnline!)^24,25^ may experience larger net desirable consequences and provide a more favourable cost-effectiveness profile (Fig. 1). On the other hand, women with low clinical risk may experience smaller or no net desirable consequences. Indirect evidence from the MINDACT trial using the 70-GS supports that conclusion. In fact, in this trial there are very small, if any, benefits from chemotherapy in low clinical risk women, independently of the genomic risk.^6^

New relevant results have been published on the 21-RS since the systematic review used for this recommendation was conducted.^45,46^ Recent data from the TAILORx trial stratified by age and clinical risk have been published.^45^ The authors suggest that women below 50 with an intermediate genomic risk score could have a benefit from adding chemotherapy to endocrine therapy if their clinical risk is high but the absence of a dose response and very imprecise effect estimates suggest that chance could play a major role. Furthermore, the reported analysis suggests that data for evaluating the potentially most efficient two-step testing strategy is missing.^47,48^ Mariotto and colleagues^49^ showed that application of the 21-RS risk to decide whether or not to provide chemotherapy would produce savings in the actual US real clinical practice. Another analysis using the same data indicates that savings would be much larger if testing would be performed according to a two-step strategy.^50^ The GDG, for the moment, judged that the new evidence is consistent with the recommendation.

#### 70-GS

In light of the results from the MINDACT trial,^6^ the GDG decided to split the recommendation according to clinical risk of the population under study (at low and high clinical risk, Table 5 and Fig. 1). In the low clinical risk group the GDG recommends against using the 70-GS testing to guide the use of chemotherapy (strong recommendation, very low certainty of the evidence) as there are no apparent benefits and there are very large costs (EUR 3153 per patient) (https://healthcare-quality.jrc.ec.europa.eu/sites/default/files/Guidelines/EtDs/Updated/ECIBC_GLs_EtD_70_gene_testing_low_risk.pdf).

For women with HoR-positive, HER2-negative, node-negative or up to 3 lymph nodes-positive invasive breast cancer at high clinical risk, the ECIBC GDG suggests using the 70-GS test to guide the use of chemotherapy. The judgments favoured the intervention in the high clinical risk population due to the moderate desirable effect, a balance that probably favours the use of 70-GS testing, and the large savings (Table 4). The recommendation is conditional mainly because of the low certainty of the evidence about the effects. The GDG also stated that the proportion of women with 2 or 3 positive lymph nodes was small, therefore making the results less clear in this subgroup (https://healthcare-quality.jrc.ec.europa.eu/sites/default/files/Guidelines/EtDs/Updated/ECIBC_GLs_EtD_70_gene_testing_high_risk.pdf).

## Discussion

### Statement of principal findings

The ECIBC GDG suggests the use of 21-RS in lymph node-negative women, recognising that benefits are probably larger in women at high clinical risk and suggests the use of the 70-GS only for women at high clinical risk.

### Strengths and weaknesses of the study

The strength of the recommendations includes the ECIBC’s adherence to the requirements for trustworthy development of guidelines.^51–53^ Previously, we described some limitations of our guidelines.^11,54^ The weakness of the deterministic decision tree model used is that it is, to some extent, a simplistic approach and some assumptions are questionable (i.e. negligible effects in low clinical risk, same effects in studies with different duration of follow-up). Furthermore, we did not actually quantify the side effects of chemotherapy, considering that avoiding any unnecessary chemotherapy was a desirable effect.

### Relation to other guidelines

NICE recently published guidance on multigene testing.^55^ The panel decided to evaluate the evidence for the four commercially available multigene tests included in the ECBIC question, and for the IHC4 + C test.^56,57^ The NICE guidelines did not follow the GRADE methodology and also had a different goal, i.e. deciding which tests should be funded by the UK National Health Service. The NICE 21-RS recommendation is similar to the ECIBC recommendation, a conditional recommendation limited to those patients in which the risk of distant recurrence is intermediate, using a validated tool such as PREDICT^58^ or the Nottingham Prognostic Index (Table 6). This approach is based on the assumption that in some patients the clinical and pathological prognostic parameters are consistent (low or high risk), and that multigene testing does not provide additional information. In particular, the NICE panel recommended against the use of the 70-GS, based on cost-effectiveness considerations (Table 6). Unfortunately, the NICE guideline does not allow comparison of our estimates of desirable and undesirable health effects. Some methodological differences might explain the divergent results: since real practice varies across Europe, we preferred to use theoretical scenarios as comparators and interventions not accounting for non-compliance, while the NICE model used real practice as comparator (the prevalence of chemotherapy used in this group of women in the UK), and as intervention (a change in the probability of receiving chemotherapy given the test result). Furthermore, the model used by NICE assumed that the tests are only prognostically relevant but are not predictive of the response to treatment, i.e. they calculated a constant HR of 0.77 for chemotherapy in addition to endocrine therapy compared to endocrine therapy alone in all risk groups. In contrast, the ECIBC GDG judged the benefits to be trivial or small in the low clinical risk group. Finally, for the 21-RS a commercial-in-confidence discounted test cost was used to model cost-effectiveness, while for the 70-GS the regular market price was used. It is worth noting that despite NICE stating that the major benefit of the genetic testing strategies would be a reduction of chemotherapy, the cost models predict health benefits only if chemotherapy is increased.

The American Society of Clinical Oncology (ASCO) also provided recommendations on the use of multigene tests.^59–61^ Despite a different methodological approach, the direction of the recommendations is the same, but the strength is not (Table 6). There are differences in the grading of certainty of the evidence, considered as high by the ASCO panellists, while the GDG valued the evidence as very low for the 21-RS and low for the 70-GS. Unfortunately, we were unable to deduce the details of the ASCO evidence rating approach and also the criteria and judgments that were used to determine the strength of the recommendations. Unlike us, ASCO and NICE made recommendations on the 12-MS and the PAM50-RORS to guide treatment decisions,^55,62^ mainly because they did not exclude studies based on prognostic results only.

### Meaning of the study

The implications of our recommendations are context dependent. The criteria used for making decisions on the provision or not of adjuvant treatment differ between countries. Therefore, the cost-benefit profile of introducing one of the multigene tests might also vary across countries. Decreasing costs for the tests would support a more widespread use. For these considerations, the GDG decided not to establish a threshold of recurrence risk to recommend genomic risk assessment, or a threshold for adding adjuvant chemotherapy, since these thresholds are context specific.

In conclusion, the ECIBC GDG recommendations for or against the use of 21-RS and 70-RS are justified based on the judgments made. The transparency of our approach allows understanding the rationale for making different recommendations for the two tests and risk groups.

### Unanswered questions and future research

Data protection issues may be of relevance for the 21-RS because processing of samples is centralised in one US lab and requires shipping samples abroad. Furthermore, transparent information on test results is not available and reproducibility has been questioned.^63^

The GDG recommends research on exploring in what subgroups the use of 21-RS would have larger anticipated benefits as well as carrying out longer follow-up studies for 70-GS. The recommendations will be updated according to the ECIBC monitoring strategy in place (https://healthcare-quality.jrc.ec.europa.eu/discover-ecibc/methodologies/guidelines-updating).

Furthermore, the GDG is exploring the possibility of evaluating biomarkers that may assist decision making regarding the administration of adjuvant chemotherapy on the basis of their ability to identify women with a sufficiently low risk of relapse that would allow them to be spared from chemotherapy. In contrast to the presented evidence evaluation, this would enable evidence-based and transparent recommendations based on prognostic cohort studies, randomised or not, that predict the recurrence risk of different subgroups. We are currently working on a healthcare question on the significance of Ki67 using this strategy. In a next step, such an approach might also make it possible to evaluate multigene tests in the assessment for which we found no usable evidence in the predictive search strategy used here, but for which data on the prognostic value are available.^13–17,64^

## Supplementary information

Supplementary material

## Data Availability

All data sources used during this study are described in this published article and its additional information files. The datasets analysed are available from the corresponding author on reasonable request.

## Dissemination declaration

Results will be disseminated through the ECIBC webpage (https://healthcare-quality.jrc.ec.europa.eu/european-breast-cancer-guidelines) and specific women versions of these recommendations will also be made available to facilitate access to lay people.
