# Supplementary material for: Recommendations from the European Commission Initiative on Breast Cancer for multigene testing to guide the use of adjuvant chemotherapy in patients with early breast cancer, hormone receptor positive, HER-2 negative
Source: Br J Cancer. 2021 Feb 18;124(9):1503–12. doi: 10.1038/s41416-020-01247-z (PMC8076250; doi:10.1038/s41416-020-01247-z)
Supplement: Supplementary file 1 — Supplementary material [file 41416_2020_1247_MOESM1_ESM.docx]

**Supplementary materials**

**Supplementary Table 1. Characteristics of the four multigene tests included in the present report.**

| 1. 12-gene molecular score (EndoPredict, Myriad Genetics) (Buus et al. 2016; Martin et al. 2014; Sestak et al. 2018)    - Measures the expression of 12 genes: 3 proliferation associated genes, 5 hormone receptor associated genes, 3 reference genes (normalisation), 1 control gene.    - The test can be performed in local laboratories.    - Results:      - EP score based on the gene expression      - EPclin score is calculated by adding tumour size and nodal status.      - The cut-offs for diagnostic decisions were predefined corresponding to a 10% distant recurrence rate at 10 years assuming 5 years of endocrine treatment. Patients are stratified into low- or high-risk groups. |
| --- |
| 1. 70-gene signature (MammaPrint, Agendia) (Cardoso et al. 2016)    - Measures the expression of 70 genes involved in different parts of the metastatic pathway, including growth and proliferation, angiogenesis, local invasion etc.    - It can be performed in manufacturer's central laboratory in the US and in reference laboratories located in Germany, Belgium, Spain, Canada, China, and Japan (information provided by the German agency of the manufacturer to the authors).      - Results: Discriminates in low and high genomic risk using a predefined cut-off corresponding to a 10% risk of developing distant metastases over the next 10 years without any adjuvant endocrine therapy or chemotherapy |
| 1. 21-gene recurrence score (Oncotype Dx Breast Recurrence Score, Genomic Health) (Gluz et al. 2016; Sparano et al. 2018; Sparano et al. 2015)    - Measures the expression of 21 genes: 16 cancer related genes correlated with distant recurrence-free survival, 5 reference genes (normalisation)    - Testing of samples is centralized in one manufacturer's laboratory in the US .    - Results are given as a recurrence score of between 0 and 100, used to quantify the 10 year risk of distant recurrence, assuming 5 years of endocrine treatment. Thresholds for discrimination in low, intermediate and high genomic risk have been changed because of the results of the TAILORx trial (Sparano et al. 2015; Sparano et al. 2018) |
| 1. PAM50 risk of recurrence score (Prosigna, NanoString Technologies) (Filipits et al. 2014; Gnant et al. 2014; Laenkholm et al. 2018; Sestak et al. 2018)    - It is based on the PAM50 gene signature (Filipits et al. 2014). Measures the expression of 50 genes used for the intrinsic subtype classification algorithm in addition to 8 housekeeping genes (normalisation), 5 positive controls, and 8 negative controls.    - The test can be performed in local laboratories.    - Results:      - Samples are classified into the following subtypes according to their PAM50 expression profile: luminal A, luminal B, HER2-enriched or basal-like.      - Risk of recurrence score (ROR) provides information about the risk of distant recurrence within 10 years, assuming 5 years of endocrine treatment. It is derived from an algorithm based on the PAM50 gene signature, breast cancer subtype, tumour size, nodal status and proliferation score (based on evaluation of proliferation associated genes). Definition of the thresholds for the risk groups (low, intermediate and high) depends also on the nodal status. |

**Supplementary Table 2. Search strategy for the evidence of effects**

| **Clinical question**  Should multigene tests be used in patients who have hormone receptor positive, HER-2 negative, lymph node negative or up to 3 lymph node positives invasive breast cancer to guide the use of adjuvant chemotherapy? | |
| --- | --- |
| **MEDLINE**  PubMed  31/05/2018 | **#1 "Breast Neoplasms"[Mesh] 263502**  **#2 breast[ti] 240718**  **#3 #1 OR #2 319677**  **#4 genomic assay*[tiab] 177**  **#5 recurrence score[tiab] 424**  **#6 multi-gene assay*[tiab] 16**  **#7 multigene assay*[tiab] 90**  **#8 multi-gene expression[tiab] 62**  **#9 multigene expression[tiab] 141**  **#10 prosigna[tiab] 39**  **#11 PAM50[tiab] 247**  **#12 PAM 50[tiab] 5**  **#13 50 gene[tiab] 139**  **#14 21-gene recurrence score[tiab] 467**  **#15 21 gene[tiab] 642**  **#16 70 gene signature[tiab] 197**  **#17 70 gene[tiab] 533**  **#18 endopredict[tiab] 52**  **#19 #4 OR #5 OR #6 OR #7 OR #8 OR #9 OR #10 OR #11 OR #12 OR #13 OR #14 OR #15 OR #16 OR #17 OR #18 2399**  **#20 #3 AND #19 1056** |
| **The Cochrane Library** | No performed because the question characteristics |
| **EMBASE**  Ovid Embase  31/05/2018 | **1 Breast Neoplasms/ (10201)**  **2 breast.ti. (314324)**  **3 1 or 2 (317563)**  **4 genomic assay*.ti,ab. (307)**  **5 recurrence score.ti,ab. (1202)**  **6 multi-gene assay*.ti,ab. (51)**  **7 multigene assay*.ti,ab. (173)**  **8 multi-gene expression.ti,ab. (97)**  **9 multigene expression.ti,ab. (168)**  **10 prosigna.ti,ab. (106)**  **11 PAM50.ti,ab. (622)**  **12 PAM 50.ti,ab. (35)**  **13 50 gene.ti,ab. (330)**  **14 21-gene recurrence score.ti,ab. (1308)**  **15 21 gene.ti,ab. (1281)**  **16 70 gene signature.ti,ab. (467)**  **17 70 gene.ti,ab. (795)**  **18 endopredict.ti,ab. (119)**  **19 4 or 5 or 6 or 7 or 8 or 9 or 10 or 11 or 12 or 13 or 14 or 15 or 16 or 17 or 18 (4840)**  **20 3 and 19 (2207)** |

**Supplementary table 3. Search strategy for the economic evidence**

| **Clinical question**  Should multigene tests be used in patients who have hormone receptor positive, HER-2 negative, lymph node negative or up to 3 lymph node positives invasive breast cancer to guide the use of adjuvant chemotherapy? | |
| --- | --- |
| MEDLINE  PubMed  31/05/2018 | #1 "Breast Neoplasms"[Mesh] 263502  #2 breast[ti] 240718  #3 #1 OR #2 319677  #4 genomic assay*[tiab] 177  #5 recurrence score[tiab] 424  #6 multi-gene assay*[tiab] 16  #7 multigene assay*[tiab] 90  #8 multi-gene expression[tiab] 62  #9 multigene expression[tiab] 141  #10 prosigna[tiab] 39  #11 PAM50[tiab] 247  #12 PAM 50[tiab] 5  #13 50 gene[tiab] 139  #14 21-gene recurrence score[tiab] 467  #15 21 gene[tiab] 642  #16 70 gene signature[tiab] 197  #17 70 gene[tiab] 533  #18 endopredict[tiab] 52  #19 #4 OR #5 OR #6 OR #7 OR #8 OR #9 OR #10 OR #11 OR #12 OR #13 OR #14 OR #15 OR #16 OR #17 OR #18 2399  #20 #3 AND #19 1056  #21 (Costs and Cost Analysis[MeSH Major Topic]) 206927  #22 Health Care Costs[MeSH Major Topic] 55441  #23 economic*[tiab] 225747  #24 (cost*[ti] OR cost[tiab] OR costs[tiab] OR cost effect*[tiab] OR cost utility*[tiab]) 441147  #25 (price[tiab] OR prices[tiab] OR pricing[tiab]) 31260  #26 pharmacoeconomic*[tiab] 3459  #27 budget*[tiab] 24510  #28 #21 OR #22 OR #23 OR #24 OR #25 OR #26 OR #27 730263  #29 #20 AND #28 138 |

**Supplementary figure 1. Flow chart for the evidence of effects**

**3,263 records identified from databases**

1,144 duplicates records excluded

2,098 records excluded after title/abstract screening

**22 records assessed for eligibility at full text**

**17 full-text articles excluded**

**Reasons:**

- Poster abstracts: 4
- Report only data of prognosis: 7
- Compare two different chemotherapy schemes: 4
- Data from the same RCT: 2

5 primary studies including:

- 2 marked-based design RCTs
- 2 interaction design RCTs
- 1 pooled individual data analysis from previous validation studies.

**Supplementary figure 2. Flow chart for the economic evidence**

**138 records identified from databases**

134 records excluded after title/abstract screening

**4 full-text articles excluded**

**Reasons:**

- Systematic reviews used as source of primary studies: 2
- Included only a subgroup of the study population: 1
- Did not report separate results for lymph node negative patients: 1

**4 records assessed for eligibility at full text**

**Primary studies identified from the systematic reviews: 12**

**12 primary studies:**

7 21-gene recurrence score lymph-node negative

3 21-gene recurrence score lymph-node positive N 1-3

1 70 gene signature lymph-node negative

1 both 21gene recurrence and 70 gene signature lymph-node negative

**Supplementary Figure 3**. Scenarios for the testing strategies (being the interventions, panel a) and the comparators (panel b) according to the PICO format.

Distribution of the risk groups in the populations are derived from the TAILORx trial (recruited before 2008) for the 21-gene recurrence score and from the MINDACT trial for the 70 gene signature (Cardoso 2016; Sparano 2018). The proportion of women, who do not receive chemotherapy in the comparator scenario 1 corresponds to the proportion of women in the study chemotherapy arm of the TAILORx trial who did not receive chemotherapy (Sparano 2018).


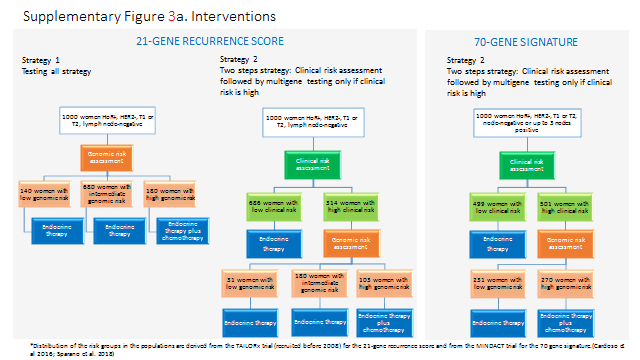


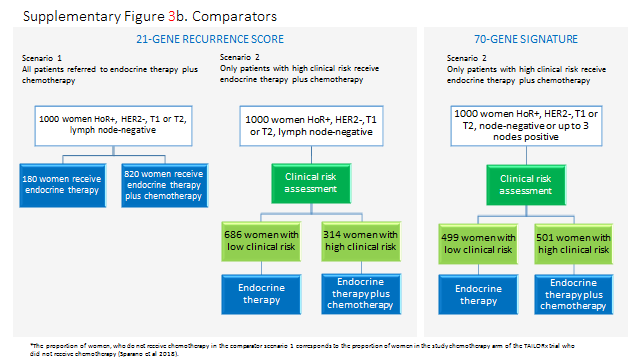


**Supplementary table 4.** Characteristics of excluded studies

| **Evidence of effects** | | |
| --- | --- | --- |
| **Excluded studies** | | |
| **Author** | **Year** | **Reason for exclusion** |
| Mamounas. | 2010 | Retrospective subgroup analysis from NSABP B-14 (tamoxifen vs placebo) and B-20 (tamoxifen + chemotherapy vs tamoxifen alone) trials. The authors combine the tamoxifen arms from the two studies with questionable assumptions. |
| Dowsett. | 2010 | Retrospective subgroup analysis from ATAC trial (anastrazole + tamoxifen vs tamoxifen). This study no includes one arm of chemotherapy + endocrine therapy. |
| Gluz. | 2016 | Retrospective subgroup analysis from PlanB trial (anthracycline-containing vs anthracycline-free chemotherapy). This study no includes one arm of chemotherapy + endocrine therapy or endocrine therapy alone. |
| Habel. | 2006 | Case-control study. Validation study |
| Mook. | 2009 | Retrospective analysis from a cohort. Validation Study. |
| Nitz. | 2017 | Retrospective analysis from PlanB trial (anthracycline-containing vs anthracycline-free chemotherapy). This study no includes one arm of chemotherapy + endocrine therapy or endocrine therapy alone. |
| Tang. | 2011 | Duplicate information (same analysis of Paik 2006) |
| Stemmer. | 2017 | Retrospective analysis from a cohort. Validation Study. |
| Stemmer. | 2017 | Retrospective analysis from a cohort. Validation Study. |
| Stravers. | 2010 | Retrospective analysis from a cohort of patients treated with chemotherapy intervention, no control group of endocrine therapy. |
| Brase. | 2011 | Poster session abstract |
| Mamounas. | 2013 | Poster session abstract |
| Mamounas. | 2017 | Retrospective subgroup analysis from NSABP B-28 trial, which compare two different chemotherapy regimens (doxorubicin/cyclophosphamide + tamoxifen versus doxorubicin/cyclophosphamide/paclitaxel + tamoxifen), no endocrine therapy arm. This study reports prognosis of Loco regional recurrence. |
| Whitworth. | 2017 | Analysis of prospective cohort of patients treated with chemotherapy intervention or endocrine therapy. The intervention allocation was no random. |
| Dubsky. | 2018 | Poster session abstract |
| Penault-Llorca. | 2018 | Retrospective subgroup analysis from PACS-01 trial, which compare two different chemotherapy regimens (FEC 100 + tamoxifen versus FEC D + tamoxifen). This study no includes one arm of only endocrine therapy. |
| Yamamoto. | 2018 | Poster session abstract |

| **Economic evidence** | | |
| --- | --- | --- |
| **Excluded studies** | | |
| **Author** | **Year** | **Reason for exclusion** |
| Blok. | 2018 | Systematic review used as source of primary studies |
| Lux. | 2018 | Did not report separate results for lymph node negative patients. The study reported results for the overall group of women with 0 to 3 lymph nodes. |
| Martinez Del Prado. | 2018 | Included only a subgroup of the study population. 1) Operable early-stage BC and adequate surgery performed for primary tumour; 2) ER+/HER2−; and 3) Pathological TNM (TNM): pT1b, if at least 2 of the following 3 factors were present: histologic grade (G) III, Ki-67 ≥14% and lymphovascular invasion; pT1c, all except those with both GI and Ki-67 <14%; pT2, all except those with GIII; pN0 or 1mi; and M0. |
| Wang. | 2018 | Systematic review used as source of primary studies |

**Supplementary table 5. Overview of included primary studies.**

| **Author (year)** | **Country** | **Design** | **Inclusion/exclusion criteria** | **Nº patients** | **Age mean (range or SD)** | **Outcome** | |
| --- | --- | --- | --- | --- | --- | --- | --- |
| **21-GENE RECURRENCE SCORE** | | | | | | | |
| **Marker-based strategy** | | | | | | | |
| Sparano 2018 | United States; Australia, Canada, Ireland, New Zeland, Peru, Puerto Rico and United Kingdom. | Prospective randomised clinical trial (open label) with parallel assignment | Inclusion:  HR +, HERB -, node negative breast cancer.  Tumour size 1.1-5.0 cm  Patients with 21-gene test result available.  Exclusion:  Previously 21-gene recurrence score DX Assay  Patients with chronic obstructive pulmonary disease requiring treatment; chronic liver disease; previous history of a cerebrovascular accident; history of congestive heart failure or other cardiac disease that would represent a contraindication to the use of an anthracycline; chronic psychiatric condition or other condition that would impair compliance with the treatment regimen. | N: 6907 patients with a midrange score of 11 to 25 underwent randomization.  Experimental: endocrine therapy with tamoxifen, anastrozole, letrozole, or exemestane (oral route) for up to 5 years  Control: standard combination chemotherapy at the discretion of the treating physician. Within 4 weeks after the last dose of chemotherapy, patients receive hormonal therapy as in the experimental group | Experimental: 55 (23-75)  Control: 55 (25-75) | Invasive disease–free survival (defined as freedom from invasive disease recurrence, second primary cancer, or death) | |
| **Treatment interaction design** | | | | | | | |
| Paik 2006 | United States | Retrospective subgroup analysis from B20 trial (Chemotherapy + endocrine therapy vs endocrine therapy alone). | Inclusion:  HR +, node negative.  Patients with 21-gene test result available.  Exclusion:  insufficient tumour (<5%of the overall tissue) as assessed by histopathology,  insufficient RNA (< 0.5 ug), or  weak RT-PCR signal (average cycle threshold for the reference genes > 35) | N: 651 patients with breast cancer.  Experimental: Chemotherapy (MFT or CMFT) + tamoxifen, n= 424 of 1529 chemotherapy-treated (27.7%)  Control: Tamoxifen alone, n=227 of 770 tamoxifen-treated (29.4%) | HER2(-): NR | Freedom from distant recurrence | |
| Albain 2010 | United States | Retrospective subgroup analysis from phase III trial S8814 (Chemotherapy + tamoxifen vs tamoxifen alone). | Inclusion:  HR +, node positive.  Patients with 21-gene test result available.  Exclusion:  insufficient tumour (<5%of the overall tissue) as assessed by histopathology,  insufficient RNA (< 0.5 ug), or  weak RT-PCR signal (average cycle threshold for the reference genes > 35) | N: 367 postmenopausal patients with breast cancer.  Experimental: Chemotherapy (CAF) + tamoxifen, n=219 of 562 chemotherapy-treated (38.9%)  Control: Tamoxifen alone, n= 148 of 354 tamoxifen-treated (41.8%) | Age: 60.4  1-3 nodes (+): 61.9%  ER(+): 96.7%  HER2(-): 88.3% | Disease-free survival | |
| **70 GENE SIGNATURE** | | | | | | | |
| **Marker-based strategy** | | | | | | | |
| Cardoso 2016 | Europe (9 European countries) | Prospective randomised clinical trial (open label) with parallel assignment | Inclusion:  Histologically confirmed unilateral primary invasive breast cancer (stage T1 or T2 or operable T3), lymph-node–negative operable disease (the protocol was revised to allow the enrolment of women with up to three positive axillary nodes).  Patients eligible for inclusion in the endocrine therapy randomization must be HR +, endocrine-responsive disease.  Exclusion:  Serious cardiac illness or medical condition  No prior neoadjuvant chemotherapy, neoadjuvant endocrine therapy, or radiotherapy for primary breast cancer | N: 2187 patients with discordant genomic risk 70-gene signature and Adjuvant! Online underwent randomization.  Experimental: Chemotherapy  Control: NO chemotherapy | Overall:  55 (23-71)  High clinical/Low genomic risk:  N+(1-3):47.2%  HR(+):98.1%  HER2(-):91.8%  Low clinical/High genomic risk:  N+(1-3): 2.5%  HR(+): 90.4%  HER2(-):87.5% | | Survival without distant metastasis (event-free rate at 5 years), as the time to first distant metastatic recurrence or death to any cause |
| **Treatment interaction design** | | | | | | | |
| Knauer 2010 | Netherlands | A pooled database from seven previously report studies with known adjuvant treatment status | Inclusion  Unilateral stage pT1-3 N0-1, M0 invasive breast carcinoma diagnosed between 1984 and 2006 | N: 541 patients’ wit 0-3 lymph node positive.  Endocrine therapy: 315 (58%)  Chemotherapy plus endocrine therapy: 226 (42%) | Age: 43% was >50 years  N0: 49%  ER (+): 90%  PR (+): 69%  HER2(-): 89% | | Breast cancer specific survival  Distant free survival |

**Supplementary table 6. Summary of findings for the 21-gene recurrence score**

| **Outcomes** | **№ of participants (studies) Follow up** | **Certainty of the evidence (GRADE)** | **Relative effect (95% CI)** | **Anticipated absolute effects^*^ (95% CI)** | |
| --- | --- | --- | --- | --- | --- |
|  |  |  |  | Risk with endocrine therapy alone | Risk difference with endocrine therapy plus chemotherapy |
| **Treatment interaction studies, lymph node negative (Paik 2006)** | | | |  | |
| **Freedom from distant recurrence –low genomic risk group** | 488 (1 RCT, Paik 20) | ⨁◯◯◯ VERY LOW^a^ | **HR 1.31** (0.46 to 3.78) | Study population | |
|  |  |  |  | 31 per 1,000 | **9 more per 1,000** (17 fewer to 82 more) |
| **Freedom from distant recurrence – intermediate genomic risk group** | 179 (1 RCT) | ⨁◯◯◯ VERY LOW^a^ | **HR 0.61** (0.24 to 1.59) | Study population | |
|  |  |  |  | 90 per 1,000 | **34 fewer per 1,000** (67 fewer to 49 more) |
| **Freedom from distant recurrence –high genomic risk group** | 211 (1 RCT) | ⨁⨁◯◯ LOW ^b^ | **HR 0.26** (0.13 to 0.53) | Study population | |
|  |  |  |  | 396 per 1,000 | **273 fewer per 1,000** (333 fewer to 162 fewer) |
| **Treatment interaction studies, 1-3 lymph node positive (Albain 2010)** | | | |  | |
| **Disease free survival** | 488 (1 RCT) | ⨁◯◯◯ VERY LOW ^c^ | - | An interaction between risk score and efficacy of chemotherapy.  low genomic risk group HR=1.02 (95% CI 0.54–1.93); intermediate risk HR = 0.72 (95%CI high 0.39-1.31); high risk HR=0.59 (95% CI 0.35–1.01); | |
| **Marker-based strategy , intermediate risk patients, RS 11-25 (Sparano 2018)** | | | |  | |
| **Invasive disease free survival** | 6712 (1 RCT) | ⨁⨁◯◯ LOW^d^ | **HR 1.14** (0.99 to 1.31) | Study population | |
|  |  |  |  | 153 per 1,000 | **19 more per 1,000** (1 fewer to 43 more) |
| **Distant recurrence free survival** | 6712 (1 RCT) | ⨁⨁◯◯ LOW^e^ | **HR 1.03** (0.80 to 1.33) | Study population | |
|  |  |  |  | 71 per 1,000 | **2 more per 1,000** (14 fewer to 22 more) |
| **Distant/local recurrence free survival** | 6712 (1 RCT) | ⨁⨁◯◯ LOW^e^ | **HR 1.12** (0.91 to 1.38) | Study population | |
|  |  |  |  | 50 per 1,000 | **6 fewer per 1,000** (4 fewer to 18 more) |
| **Overall survival** | 6712 (1 RCT) | ⨁⨁◯◯ LOW ^e^ | **HR 0.97** (0.78 to 1.21) | Study population | |
|  |  |  |  | 62 per 1,000 | **2 fewer per 1,000** (13 fewer to 12 more) |

a. Evidence was downgraded for: risk of bias (the original trial (B-20) did not provide information about the HER-2 status among the included patients; part of the sample was previously used to validate a previous version of the gene markers test which might lead to overfitting in the subsequent analysis); imprecision (there were a low number of events in each genomic risk group); indirectness (the study enrolled patients that were treated more than ten years ago, the chemotherapy regime is very different now; the study design used did not provide the number of chemotherapies avoided).

b. Evidence for this outcome was downgraded for all the reasons reported in footnote a, except imprecision.

c. Evidence was downgraded for: risk of bias (part of the sample was previously used to validate a previous version of the gene markers test which might lead to overfitting in the subsequent analysis); imprecision (there were a low number of events in each genomic risk group); indirectness (the study enrolled patients that were treated more than ten years ago, the chemotherapy regime is very different now; the study design used did not provide the number of chemotherapies avoided; a 12% of the included subjects in the analysis were HER2-positive). Additionally, an interaction analysis of the linear RS adjusted by the number of positive nodes showed a p value=0.053 for DFS. However, this effect was not constant over time. Results were not provided by strata of the number of positive lymph nodes (ie.1 to 3 vs 4 or more), instead as adjusted estimations by number of nodes.

d. Evidence was downgraded for: indirectness (a different threshold was used for interpreting the test`s results, thus, the intermediate range was defined as those with a score from 11 to 25, which is not consistent with previous studies assessing the same test); risk of bias (the study did not use an appropriate non-inferiority design; additionally, the high rate of non-adherence lead to an increase of the sample size non-initially planned; there was an important imbalance in the proportions of patients that broke the protocol, which was larger in the chemotherapy plus endocrine therapy group); imprecision (the confidence interval of the effect sizes was wide, indicating a potential harmful effect for endocrine therapy alone for the upper limit)

e. Evidence for this outcome was downgraded for all the reasons reported in footnote d, except imprecision.

**Supplementary table 7. Summary of findings for the 70-gene signature**

| **Outcomes** | | **№ of participants (studies) Follow up** | **Certainty of the evidence (GRADE)** | | | **Relative effect (95% CI)** | **Anticipated absolute effects^*^ (95% CI)** | | |
| --- | --- | --- | --- | --- | --- | --- | --- | --- | --- |
|  |  |  |  |  |  |  | **Risk with endocrine therapy alone** | | **Risk difference with chemotherapy plus endocrine therapy** |
| **Treatment interaction studies, lymph node negative or up to 3 lymph nodes positive(Knauer 2010)** | | | | | | |  | | |
| Distant free survival –low genomic risk group | | 252 (1 observational study)^a^ | ⨁◯◯◯ VERY LOW^b^ | | | **HR 0.26** (0.03 to 2.02) | Study population | | |
|  |  |  |  |  |  |  | 69 per 1,000 | | **51 fewer per 1,000** (67 fewer to 65 more) |
| Distant free survival –high genomic risk group | | 289 (1 observational study)^a^ | ⨁◯◯◯ VERY LOW^b^ | | | **HR 0.35** (0.17 to 0.71) | Study population | | |
|  |  |  |  |  |  |  | 241 per 1,000 | | **149 fewer per 1,000** (195 fewer to 63 fewer) |
| Breast cancer specific survival –low genomic risk group | | 252 (1 observational study)^a^ | ⨁◯◯◯ VERY LOW^b^ | | | **HR 0.58** (0.07 to 4.98) | Study population | | |
|  |  |  |  |  |  |  | 29 per 1,000 | | **12 fewer per 1,000** (27 fewer to 106 more) |
| Breast cancer specific survival –high genomic risk group | | 289 (1 observational study)^a^ | ⨁◯◯◯ VERY LOW^b^ | | | **HR 0.21** (0.07 to 0.59) | Study population | | |
|  |  |  |  |  |  |  | 191 per 1,000 | | **148 fewer per 1,000** (177 fewer to 74 fewer) |
| **Marker-based strategy, patients with low clinical risk and high genomic risk score and patients with high clinical risk and low genomic risk score (Cardoso 2016)** | | | | | | |  | | |
| Distant metastases free survival | 1228 (1 RCT)^c^ | | | ⨁⨁◯◯ VERY LOW^d^ | **HR 0.65** (0.38 to 1.10) | | Study population | | |
|  |  |  |  |  |  |  | 58 per 1,000 | **20 fewer per 1,000** (36 fewer to 6 more) | |
| Disease free survival | 1228 (1 RCT)^c^ | | | ⨁⨁◯◯ LOW^d^ | **HR 0.64** (0.43 to 0.95) | | Study population | | |
|  |  |  |  |  |  |  | 104 per 1,000 | **36 fewer per 1,000** (58 fewer to 5 fewer) | |
| Overall survival | 1228 (1 RCT)^c^ | | | ⨁⨁◯◯ LOW^d^ | **HR 0.63** (0.29 to 1.37) | | Study population | | |
|  |  |  |  |  |  |  | 28 per 1,000 | **10 fewer per 1,000** (20 fewer to 10 more) | |

a. Individual patient pooled analysis from previous reported studies (Knauer 2010)

b. Evidence was downgraded for: risk of bias (follow-up was censored at 5 years, the follow-up time might be short to assess of the outcomes of interest; data included in the patient data pooled analysis were previously used in validation studies); indirectness (results were not provided by strata of negative and positive lymph nodes (up to 3), instead as adjusted estimations); imprecision (there were a low number of events in each genomic risk group and low power to test interaction).

c. The MINDACT (Cardoso 2016) trial aims to assess non-inferiority of the arm with endocrine therapy only vs. chemotherapy plus endocrine therapy in the group of high clinical (according to AdjuvantOnnline! (Ravdin 2001; Olivotto 2005) low genomic rsik. We report here the per-protocol analysis which is a more conservative approach in this context.

d. Evidence was downgraded for: risk of bias (results were reported at 5 years. This follow-up time might be insufficient for the measured outcomes; there was imbalance between groups in the proportions of violations to the protocol; additionally, a group of patients were incorrectly classified by test) indirectness (The study enrolled patients that were treated more than ten years ago, the chemotherapy regime is very different now; the study design used did not provide the number of chemotherapies avoided; the population was defined as High clinical Risk by the Adjuvant Online!: -Lymph node negative: 52%; HR +: 98%; HER2 -: 92%; Size >2cm: 58%); imprecision (there were a low number of events in each genomic risk group).

**Supplementary table 8. Definition of the low and high clinical risk According to Adjuvant! Online (version 8.0 with HER2 Status) described in detail in the MINDACT trial (Cardoso 2016).**

| **Low clinical risk** refers to those patients with HR-positive and HER2-negative invasive breast cancer with either:   - G1 and node negative and tumour size </= 3cm - G1 and 1-3 positive nodes and tumour size </= 2cm - G2 and node negative and tumour size </= 2cm - G3 and node negative and tumour size </= 1cm. | **High clinical risk** refers to those patients with HR-positive and HER2-negative invasive breast cancer with either:   - G1 and node negative and tumour size 3.1-5 cm - G1 and 1-3 positive nodes and tumour size 2.1-5 cm - G2 and node negative and tumour size 2.1-5 cm - G2 and 1-3 positive nodes and any tumour size - G3 and node negative and tumour size 2.1-5 cm - G3 and 1-3 positive nodes and any tumour size. |
| --- | --- |

**References**

- Albain KS, Barlow WE, Shak S, Hortobagyi GN, Livingston RB, Yeh IT et al. Breast Cancer Intergroup of North America. Prognostic and Predictive Value of the 21-Gene Recurrence Score Assay in a Randomized Trial of Chemotherapy for Postmenopausal, Node-Positive, Estrogen Receptor-Positive Breast Cancer. Lancet Oncol 11:55-65, 2010.
- Blok EJ, Bastiaanet E, van den Hout WB, Liefers GJ, Smit VTHBM, Kroep JR et al. Systematic review of the clinical and economic value of gene expression profiles for invasive early breast cancer available in Europe. Cancer Treat Rev 62:74-90, 2018.
- Buus R, Sestak I, Kronenwett R, Denkert C, Dubsky P, Krappmann K et al. Comparison of EndoPredict and EPclin With Oncotype DX Recurrence Score for Prediction of Risk of Distant Recurrence After Endocrine Therapy. J Natl Cancer Inst. 2016 Jul 10;108(11).
- Cardoso F, van't Veer LJ, Bogaerts J, Slaets L, Viale G, Delaloge S. et al. 70-Gene Signature as an Aid to Treatment Decisions in Early-Stage Breast Cancer. N Engl J Med 375:717-29, 2016.
- Dowsett M, Cuzick J, Wale C, Forbes J, Mallon EA, Salter J et al. Prediction of risk of distant recurrence using the 21-gene recurrence score in node-negative and node-positive postmenopausal patients with breast cancer treated with anastrozole or tamoxifen: a TransATAC study. J Clin Oncol 28:1829-34, 2010.
- Dubsky PC, Fesl C, Singer CF, Egle D, Wette V, Petru E et al. Abstract GS6-04: The EndoPredict score predicts residual cancer burden after neoadjuvant chemotherapy and after neoendocrince therapy in HR+/HER2- breast cancer patients from ABCSG 34. Cancer Res 78 (4 Supplement):GS6-04, 2018.
- Filipits M, Nielsen TO, Rudas M, Greil R, Stöger H, Jakesz R et al. The PAM50 risk-of-recurrence score predicts risk for late distant recurrence after endocrine therapy in postmenopausal women with endocrine-responsive early breast cancer. Clin Cancer Res 20:1298-305, 2014.
- Gluz O, Nitz UA, Christgen M, Kates RE, Shak S, Clemens M et al. West German Study Group Phase III PlanB Trial: First Prospective Outcome Data for the 21-Gene Recurrence Score Assay and Concordance of Prognostic Markers by Central and Local Pathology Assessment. J Clin Oncol 34:2341-9, 2016.
- Gnant M, Filipits M, Greil R, Stoeger H, Rudas M, Bago-Horvath Z et al. Predicting distant recurrence in receptor-positive breast cancer patients with limited clinicopathological risk: using the PAM50 Risk of Recurrence score in 1478 postmenopausal patients of the ABCSG-8 trial treated with adjuvant endocrine therapy alone. Ann Oncol 25:339-45, 2014.
- Habel LA, Shak S, Jacobs MK, Capra A, Alexander C, Pho M et al. A population-based study of tumor gene expression and risk of breast cancer death among lymph node-negative patients. Breast Cancer Res 8:R2, 2006.
- Knauer M, Mook S, Rutgers EJ, Bender RA, Hauptmann, van de Vijver MJ et al. The predictive value of the 70-gene signature for adjuvant chemotherapy in early breast cancer. Breast Cancer Res Treat 120:655-61, 2010.
- Lænkholm AV, Jensen MB, Eriksen JO, Bruun Rasmussen B, Knoop AS, Buckingham W et al. PAM50 Risk of Recurrence Score Predicts 10-Year Distant Recurrence in a Comprehensive Danish Cohort of Postmenopausal Women Allocated to 5 Years of Endocrine Therapy for Hormone Receptor-Positive Early Breast Cancer. J Clin Oncol 36:735-740, 2018.
- Lux MP, Nabieva N, Hildebrandt T,Rebscher H, Kümmel S, Blohmer JU et al. Budget impact analysis of gene expression tests to aid therapy decisions for breast cancer patients in Germany. Breast 37:89-98, 2018.
- Mamounas EP, Liu Q, Paik S, Baehner FL, Tang G, Jeong JH et al. 21-Gene Recurrence Score and Locoregional Recurrence in Node-Positive/ER-Positive Breast Cancer Treated With Chemo-Endocrine Therapy. J Natl Cancer Inst 109:4, 2017.
- Mamounas EP, Tang G, Fisher B, Paik S, Shak S, Costantino JP et al. Association between the 21-gene recurrence score assay and risk of locoregional recurrence in node-negative, estrogen receptor-positive breast cancer: results from NSABP B-14 and NSABP B-20. J Clin Oncol 28:1677-83, 2010.
- Mamounas TP. Predicting locoregional recurrence after neoadjuvant hemotherapy in patients with breast cancer. Clin Adv Hematol Oncol 11:175-7, 2013.
- Martin M, Brase JC, Calvo L, Krappmann K, Ruiz-Borrego M, Fisch K et al. Clinical validation of the EndoPredict test in node-positive, chemotherapy-treated ER+/HER2- breast cancer patients: results from the GEICAM 9906 trial. Breast Cancer Res 16:R38, 2014.
- Martínez Del Prado P, Alvarez-López I, Domínguez-Fernández S, Plazaola A, Ibarrondo O, Galve-Calvo E et al. Clinical and economic impact of the 21-gene recurrence score assay in adjuvant therapy decision making in patients with early-stage breast cancer: pooled analysis in 4 Basque Country university hospitals. Clinicoecon Outcomes Res 10:189-199, 2018.
- Mook S, Schmidt MK, Viale G, Pruneri G, Eekhout I, Floore A et al. The 70-gene prognosis-signature predicts disease outcome in breast cancer patients with 1–3 positive lymph nodes in an independent validation study. Breast Cancer Res Treat 116:295–302, 2009.
- Nitz U, Gluz O, Christgen M, Kates RE, Clemens M, Malter W et al. Reducing chemotherapy use in clinically high-risk, genomically low-risk pN0 and pN1 early breast cancer patients: five-year data from the prospective, randomised phase 3 West German Study Group (WSG) PlanB trial. Breast Cancer Res Treat 165:573-583, 2017.
- Olivotto IA, Bajdik CD, Ravdin PM, Speers CH, Coldman AJ, Norris BD et al. Population-based validation of the prognostic model ADJUVANT! for early breast cancer. J Clin Oncol 23: 2716-25, 2005.
- Paik S, Tang G, Shak S, Kim C, Baker J, Kim W et al. Gene expression and benefit of chemotherapy in women with node-negative, estrogen receptor-positive breast cancer. J Clin Oncol 24:3726-34, 2006.
- Penault-Llorca F, Filleron T, Asselain B, Baehner FL, Fumoleau P, Lacroix-Triki M et al. The 21-gene Recurrence Score® assay predicts distant recurrence in lymph node-positive, hormone receptor-positive, breast cancer patients treated with adjuvant sequential epirubicin- and docetaxel-based or epirubicin-based chemotherapy (PACS-01 trial). BMC Cancer 18:526, 2018.
- Ravdin PM, Siminoff LA, Davis GJ, Mercer MB, Hewlett J, Gerson N et al. Computer program to assist in making decisions about adjuvant therapy for women with early breast cancer. J Clin Oncol 19: 980-91, 2001.
- Rupp AK, Rupp C, Keller S, Brase JC, Ehehalt R, Fogel M et al. Loss of EpCAM expression in breast cancer derived serum exosomes: role of proteolytic cleavage. Gynecol Oncol 122:437-46, 2011.
- Sestak I, Buus R, Cuzick J, Dubsky P, Kronenwett R, Denkert C et al. Comparison of the Performance of 6 Prognostic Signatures for Estrogen Receptor-Positive Breast Cancer: A Secondary Analysis of a Randomized Clinical Trial. JAMA Oncol 4:545-553, 2011.
- Sparano JA, Gray RJ, Makower DF, Pritchard K, Albain KS, Hayes DF et al. Adjuvant Chemotherapy Guided by a 21-Gene Expression Assay in Breast Cancer. N Engl J Med 379:111-121, 2018.
- Sparano JA, Gray RJ, Makower DF, Pritchard K, Albain KS, Hayes DF et al. Prospective Validation of a 21-Gene Expression Assay in Breast Cancer. N Engl J Med 373:2005-14, 2015.
- Stemmer SM, Steiner M, Rizel S, Geffen DB, Nisenbaum B, Peretz T et al. Clinical outcomes in ER+ HER2-node-positive breast cancer patients who were treated according to the Recurrence Score results: evidence from a large prospectively designed registry. NPJ Breast Cancer 3:32, 2017.
- Stemmer SM, Steiner M, Rizel S, Soussan-Gutman L, Ben-Baruch N, Bareket-Samish A et al. Clinical outcomes in patients with node-negative breast cancer treated based on the recurrence score results: evidence from a large prospectively designed registry. NPJ Breast Cancer 3:33, 2017.
- Straver ME, Glas AM, Hannemann J, Wesseling J, van de Vijver MJ, Th Rutgers EJ et al. The 70-gene signature as a response predictor for neoadjuvant chemotherapy in breast cancer. Breast Cancer Res Treat ;119:551-8, 2010.
- Tang G, Shak S, Paik S, Anderson SJ, Costantino JP, Geyer CE et al. Comparison of the prognostic and predictive utilities of the 21-gene Recurrence Score assay and Adjuvant! for women with node-negative, ER-positive breast cancer: results from NSABP B-14 and NSABP B-0. Breast Cancer Res Treat 127:133-42, 2011.
- Wang SY, Dang W, Richman I, Mougalian SS, Evans SB, Gross CP. Cost-effectiveness analyses of the 21-gene assay in breast cancer: systematic review and critical appraisal. J Clin Oncol 36:1619-1627, 2018.
- Whitworth P, Beitsch P, Mislowsky A, Pellicane JV, Nash C, Murray M et al. Chemosensitivity and Endocrine Sensitivity in Clinical Luminal Breast Cancer Patients in the Prospective Neoadjuvant Breast Registry Symphony Trial (NBRST) Predicted by Molecular Subtyping. Ann Surg Oncol 24:669-675, 2017.
- Yamamoto Y, Iwata H, Masuda M, Fujisawa T, Toyama T, Kashiwaba M et al. Abstract PD5-03: TransNEOS: Validation of the oncotype DX recurrence score (RS) testing core needle biopsy samples from NEOS as predictor of clinical response to neoadjuvant endocrine therapy for postmenopausal estrogen receptor positive (ER+), HER2 negative (HER2-) breast cancer patients. Cancer Res 78(4 Supplement):PD5-03, 2018.
